# Annual and spatial variation in composition and activity of terrestrial mammals on two replicate plots in lowland forest of eastern Ecuador

**DOI:** 10.7717/peerj.4241

**Published:** 2018-01-09

**Authors:** John G. Blake, Bette A. Loiselle

**Affiliations:** 1 Department of Wildlife Ecology and Conservation, University of Florida, Gainesville, FL, United States of America; 2 Department of Wildlife Ecology & Conservation and Center for Latin American Studies, University of Florida, Gainesville, FL, United States of America

**Keywords:** Camera trap, Ecuador, Lowland forest, Mammal, Spatial variation, Temporal variation

## Abstract

Terrestrial mammals are important components of lowland forests in Amazonia (as seed dispersal agents, herbivores, predators) but there are relatively few detailed studies from areas that have not been affected by human activities (e.g., hunting, logging). Yet, such information is needed to evaluate effects of humans elsewhere. We used camera traps to sample medium to large-sized terrestrial mammals at a site in lowland forests of eastern Ecuador, one of the most biologically rich areas in the world. We deployed cameras on two study plots in *terra firme* forest at Tiputini Biodiversity Station. Sixteen cameras were arranged 200 m apart in a 4 × 4 grid on each plot. Cameras were operated for  60 days in January–March, 2014–2017, for a total of 3,707 and 3,482 trap-days on the two plots (Harpia, Puma). A total of 28 species were recorded; 26 on Harpia and 25 on Puma. Number of species recorded each year was slightly greater on Harpia whereas overall capture rates (images/100 trap-days) were higher on Puma. Although most species were recorded on each plot, differences in capture rates meant that yearly samples on a given plot were more similar to each other than to samples on the other plot. Images of most species showed a clumped distribution pattern on each plot; *Panthera onca* was the only species that did not show a clumped distribution on either plot. Images at a given camera location showed no evidence of autocorrelation with numbers of images at nearby camera locations, suggesting that species were responding to small-scale differences in habitat conditions. A redundancy analysis showed that environmental features within 50 or 100 m of camera locations (e.g., elevation, variation in elevation, slope, distance to streams) accounted for significant amounts of variation in distribution patterns of species. Composition and relative importance based on capture rates were very similar to results from cameras located along trails at the same site; similarities decreased at increasing spatial scales based on comparisons with results from other sites in Ecuador and Peru.

## Introduction

Mammals are important components of lowland Neotropical forests (Terborgh, 1988; Dirzo & Miranda, 1990) but detailed information on composition of mammal assemblages is available from relatively few sites (Voss & Emmons, 1996; Voss, Lunde & Simmons, 2001; Ahumada et al., 2011; Beaudrot et al., 2016), particularly from areas that have not been disturbed by human activities. Mammals may influence forest structure and composition through their actions as seed dispersal agents, seed predators, and herbivores (Howe & Smallwood, 1982; Terborgh et al., 1993; Wunderle, 1997; Wright, 2003; Connell et al., 2005; Muller-Landau & Hardesty, 2005; Lambert, 2011; Fleming & Kress, 2013). Similarly, large terrestrial mammals, such as *Tayassu pecari*, may alter vegetation structure and composition through activities (e.g., trampling, wallowing, digging, etc.) that physically alter the substrate (Dirzo & Miranda, 1990; J Blake, pers. obs., 2017). As predators, mammals (e.g., *Panthera onca*, *Puma concolor*, *Leopardus pardalis*) can exert top-down pressures on many other species; loss of such predators can then have a variety of cascading effects (Terborgh, 1988; Terborgh et al., 2001; but see Wright, Gompper & DeLon, 1994).

Mammals also are important sources of food for many people and hunting can have significant impacts (direct and indirect) on mammal communities, altering abundances, distribution patterns, diurnal activity, and other behaviors (Di Bitetti et al., 2008b; Effiom et al., 2014; Mayor et al., 2015; Hegerl et al., 2017) with potential impacts on the plant community (Wright, 2003). Hunting often interacts synergistically with logging (Poulsen, Clark & Bolker, 2011; Mayor et al., 2015) and other extractive activities (e.g., oil; Finer et al., 2008; Bass et al., 2010) that increase access for hunters via road construction (Suárez et al., 2009; Suárez et al., 2012). An evaluation of the effects of human activities requires studies from areas that have not been affected by humans (Blake et al., 2017) although such opportunities are increasingly hard to find (Dirzo & Miranda, 1990; Voss & Emmons, 1996).

Lowland forests of eastern Ecuador are among the most diverse anywhere (Bass et al., 2010). Although there have been several studies on bird communities in these forests (Canaday, 1997; English, 1998; Pearman, 2002; Blake, 2007; Blake & Loiselle, 2015; Blake & Loiselle, 2016), comparable studies on mammal assemblages are less common (Arguillin et al., 2010; Arguillin, Urgilés & Noss, 2010; Beaudrot et al., 2016). Yet, given the importance of mammals to tropical forest ecosystems (Dirzo & Miranda, 1990), a better understanding of how communities or assemblages vary in space and time is important from many perspectives.

Given the wide range in body sizes, behavior (e.g., diurnal vs nocturnal), abundance, and habitat use, standardized surveys of tropical mammals can be difficult. No single method is suitable for surveying the entire mammalian fauna of a site (Voss & Emmons, 1996) and as a consequence, a variety of methods have been used to sample mammal communities (or components of such communities; Voss, Lunde & Simmons, 2001). Camera traps have emerged over the last decade or two as an important tool for surveying mammals, particularly medium to large-sized (e.g., >∼0.5–1 kg) terrestrial species (Tobler et al., 2008; Espartosa, Pinotti & Pardini, 2011; Ahumada et al., 2011; Ahumada, Hurtado & Lizcano, 2013; O’Connell, Nichols & Karanth, 2011; Burton et al., 2015; Hegerl et al., 2017); camera traps also have been used to document mammal activity in the canopy of lowland forest (Gregory et al., 2014). This technique also has been used to develop inventories of mammals (Trolle, 2003a; Trolle, 2003b; Ahumada et al., 2011; Negrões et al., 2011; Gonthier & Castañeda, 2013; Mugerwa et al., 2013; Meyer et al., 2015), to assess activity patterns (Lizcano & Cavelier, 2000; Di Bitetti, Paviolo & De Angelo, 2006; Blake et al., 2012; Foster et al., 2013), to document use of specific habitats (Tobler, Carrillo-Percastegui & Powell, 2009; Blake et al., 2010; Blake et al., 2011), and to provide estimates of abundance, particularly of species with distinctive markings, such as *Panthera onca* and *Leopardus pardalis* (Maffei, Cuéllar & Noss, 2004; Karanth et al., 2006; Di Bitetti et al., 2008a; Rocha et al., 2016; Mosquera et al., 2016). Further, camera traps have been used to evaluate effects of human activities, such as roads, logging, and hunting, on mammal distribution and abundance (Di Bitetti et al., 2008b; Di Bitetti, Paviolo & De Angelo, 2014; Ahumada, Hurtado & Lizcano, 2013; Blake, Mosquera & Salvador, 2013; Meyer et al., 2015).

Assemblages of species vary in composition and abundance at different spatial and temporal scales so the scale at which organisms are sampled can affect perceptions of community structure. Habitat heterogeneity can be an important influence on species distribution and abundance patterns (Pearman, 2002; Tobler, Carrillo-Percastegui & Powell, 2009) as can temporal variation in environmental conditions (e.g., rainfall; Blake & Loiselle, 2015). Most studies that have used camera traps to sample mammal communities in tropical forests have been done over relatively large areas, often because studies are designed for large felids that may travel over long distances; consequently, cameras often are located far apart (e.g., >1–2 km; Maffei, Cuéllar & Noss, 2004; Di Bitetti, Paviolo & De Angelo, 2006; Karanth et al., 2006; Rocha et al., 2016; but see Thornton, Branch & Sunquist, 2011a; Thornton, Branch & Sunquist, 2011b). Large-scale surveys likely cover a broad range of habitats but may not reveal patterns of activity in relation to smaller-scale differences in habitat. Temporal activity and local-scale distribution patterns may reflect small-scale variation in topography, habitat, and/or presence of competitors or predators. Here, we focus on small-scale patterns of mammal activity using camera traps on two study plots located in *terra firme* forest of eastern Ecuador.

Previous studies on birds at the same study site (Blake, 2007; Blake & Loiselle, 2009; Blake & Loiselle, 2015; Blake & Loiselle, 2016) have demonstrated that overall composition of the avian assemblage is very similar on two 100-ha study plots located ∼1.5 km apart. Yet, there are significant differences in distribution and abundance of individual species, with differences related to small-scale differences in habitat conditions on the two plots. Here, we compare patterns of species richness, activity, and spatial distribution of terrestrial mammals found on these same two tracts of *terra firme* forest. Given their close proximity, conditions on the two plots almost certainly reflect similar long-term histories. Thus, we expected mammal assemblages to be broadly similar as well. Yet, given small-scale variation in habitats, topography, resources, and other factors that affect species distributions, we also expected some species to show pronounced differences in activity between plots. Cameras were located in relatively close proximity within a plot(∼200 m apart) as we were interested in small-scale differences. Thus, we do not attempt to estimate density and recognize that some individuals likely occurred at more than one camera location.

**Figure 1:**
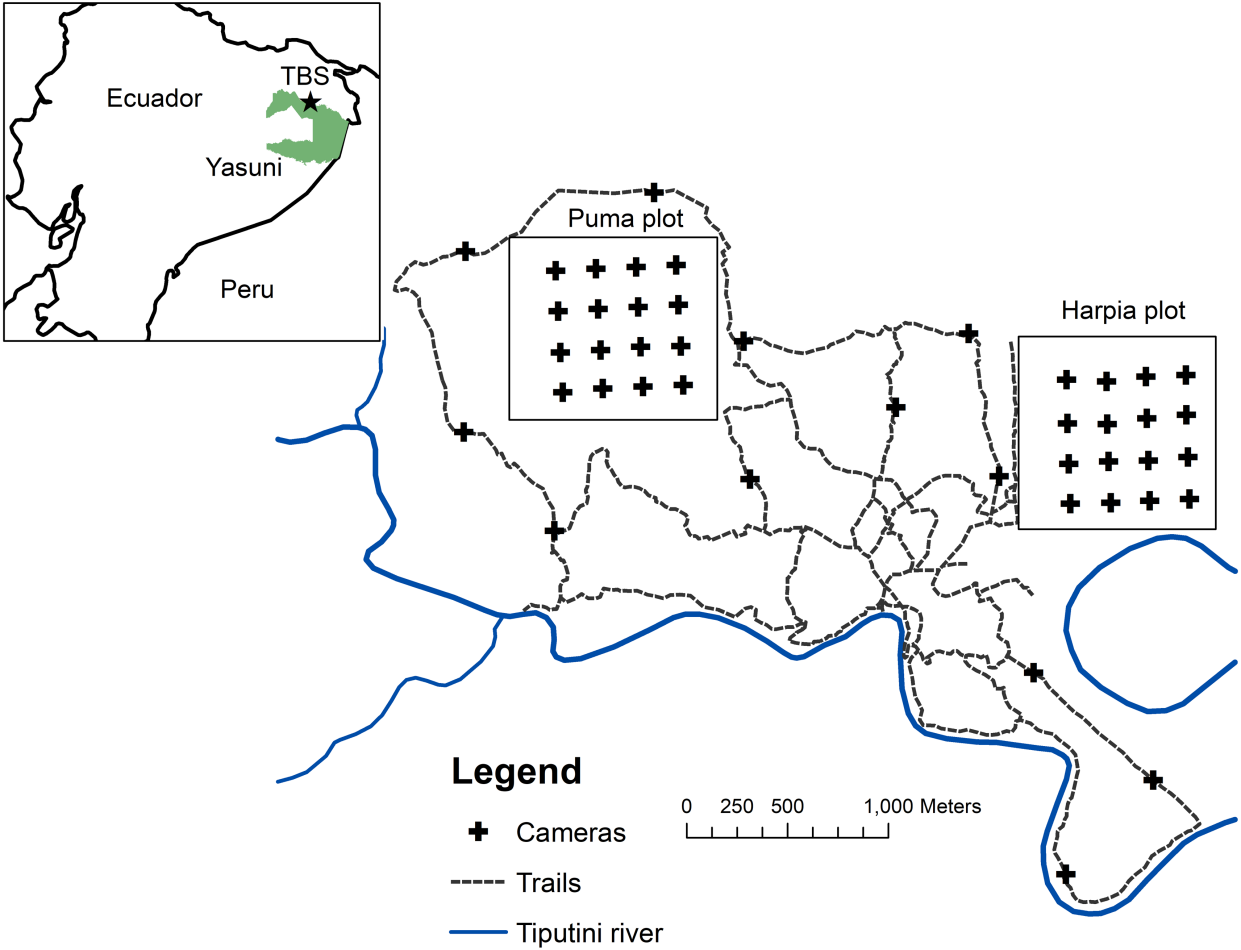
Map of camera locations at Tiputini Biodiversity Station. Locations of cameras on two 100-ha study plots at Tiputini Biodiversity Station, Ecuador. Also shown are locations of cameras along trails (see Blake, 2007).

## Methods

### Study site

Research was conducted at Tiputini Biodiversity Station (TBS), Orellana Province, Ecuador (*ca* 0°37′S, 76°10′W, ∼190–270 m above sea level). Work at Tiputini Biodiversity Station was conducted in accordance with research permit No. 0005-FAU-MAE-DPOPNY, Ministerio del Ambiente, Ecuador. TBS is located adjacent to Yasuní National Park on a tract of largely undisturbed lowland forest within the biologically diverse Yasuní Biosphere Reserve (Bass et al., 2010). The station and nearby areas are dominated by *terra firme* forest but *várzea* forest, palm swamps, and various successional habitats also are present. Mean annual precipitation at Yasuní Research Station, approximately 30 km WSW of TBS, is about 3,100 mm. Two plots (Harpia, Puma; ∼1 km × 1 km each) were established in *terra firme* forest during 2001. Both plots are gridded (100 × 200-m grid lines) and marked (Fig. 1). Transects are approximately 0.5–1 m wide and are typically cleared during November-December of each year. The Harpia plot ranges from ∼201 to 233 m elevation and is characterized by more dissected upland forest. The Puma plot has less relief overall although elevation range is similar, from ∼209 to 235 m. Both areas experience partial, temporary inundation (approximately 5 to 10 ha, depending on height of the flood) when small streams back up as the Tiputini River rises; Puma has more areas that fill with persistent standing water during the rainy season (see Loiselle et al., 2007).

### Camera traps

Cameras (Reconyx Hyperfire; Reconyx, Inc., Holmen, WI, USA) triggered by an infrared heat-and-motion detector were located ∼200 m apart in a grid of 16 cameras on each plot (Fig. 1); only one camera was placed at each location. Cameras were active from mid-January until mid-March, 2014–2017 (∼60 days/year). All cameras were set to record 5 images during each detection event, with a 1-sec delay between images. We set cameras with a minimum time of 5 min between sets of images. Cameras remained continuously activated (except when malfunctions occurred); date and time were automatically stamped on each image. Cameras were visited frequently to check batteries and camera placement (cameras were occasionally disturbed by *Tayassu pecari*). Assuming that individual animals within 100 m of the perimeter of the camera grid (i.e., half the distance between cameras) might be expected to pass in front of a camera at some point, then the 16 cameras would have covered an area of approximately 64 ha; the area sampled would be ∼100 ha if individuals within 200 m from the perimeter might be detected. Many individuals of large species (e.g., *Panthera onca*) certainly range well beyond the area covered by the camera grid; other, smaller species (e.g., *Myoprocta pratti*) may occupy areas sampled by only one camera. We calculated number of trap-days for each camera location as the time the camera was placed in operation until it was taken down or until the last image was taken (based on the date and time stamp on the images) if batteries had failed or other malfunctions had occurred. We combined records across months during each year.

Both plots were surveyed to record elevation (at 25 m intervals), locations of permanent markers, and stream courses. These data were used to produce GIS databases for permanent grid markers, streams, elevation, slope, and aspect (Loiselle et al., 2007). We used 50 and 100-m buffers around each camera to characterize environmental space around each camera location. Plots were first divided into 1 × 1-m grid cells and assigned unique numbers to each camera location. Thus, grid cells within either 50 or 100 m of each camera were identified by the same value. The environmental characteristics of each location were then determined using zonal statistics in GIS (SPATIAL ANALYST, ESRI, Redlands, CA, USA). Zonal statistics provide minimum, maximum, mean, and standard deviation of values for each environmental variable; camera identification values defined the zones. After examining correlations among variables, we retained mean and standard deviation of elevation (m), slope (degrees), and distance (Euclidean) from stream (m).

### Analyses

We summarized images by species and date. We classified images as belonging to independent records if more than 30 min elapsed between consecutive images of the same species at a given location (O’Brien, Kinnaird & Wibisono, 2003; Datta, Anand & Naniwadekar, 2008; Blake et al., 2011). We indexed activity in terms of number of images/100 trap-days (hereafter referred to as capture rates; i.e., captures of images) irrespective of the number of individuals in each image (e.g., peccaries often were represented by multiple individuals). We do not assume that such rates are a true estimate of actual abundance (Burton et al., 2015) but simply assume that, for comparative purposes, such rates provide a reasonable estimate of activity (frequency with which animals pass in front of a camera) at a given camera location (Foster, Harmsen & Doncaster, 2010; Blake et al., 2011; Meyer et al., 2015; Blake et al., 2017). Capture rates may be affected by factors that do not relate directly to abundance so interpretation of results must keep these limitations in mind. The distance between camera locations in this study is considerably less than that found in most camera-trap studies (reviewed by Burton et al., 2015) and, as consequence, images from different cameras are not necessarily independent as individuals of a variety of species, particularly larger species (e.g., *Panthera onca, Puma concolor, Leopardus pardalis, Tayassu peccari, Pecari tajacu, Tapirus terrestris*) may be recorded at more than one location (Blake et al., 2014; Blake et al., 2016). Thus, rates of capture may reflect variation in spatial activity patterns as well as variation in numbers of individuals. Differences in yearly capture rates between plots were evaluated with a paired *t*-test. We used correlation (Spearman’s rank) tests to examine the level of similarity in capture rates of species between plots within a year and between years within a plot. We also calculated an interpolated jackknife estimate of number of species present during a given sample using program SPECRICH (Hines, 1996).

Spatial distribution of individual species (i.e., clumped, random, uniform; based on numbers of images at camera locations) was evaluated using Program PASSaGE Version 2.0.10.18 (Rosenberg & Anderson, 2011). PASSaGE computes means and variances from counts (e.g., number of images/camera) and calculates a series of indices to describe patterns of variation. We used the Index of Dispersion (ID; based on the variance-to-mean ratio) as an indication of whether distribution of images among camera locations was clumped (ID > 1.0), random (ID = 1.0), or uniform (ID < 1.0). Departure from random distribution was evaluated with a chi-square test (Rosenberg & Anderson, 2011). We also used PASSaGE to test for spatial autocorrelation in distribution of individual species (i.e., correlograms; Moran’s I) to examine if number of captures of a given species at one location was correlated with captures at cameras at different distances. Moran’s I (Moran, 1950) ranges from 1 to −1 with an expected value of ∼0 for large sample sizes and no spatial autocorrelation. Significance levels of correlations were examined with permutation tests. We used Mantel correlograms to determine if species composition (based on Jaccard similarity coefficients) at camera locations exhibited spatial autocorrelation depending on distances among cameras. We also used Mantel tests to examine whether the compositional similarity (all common species) between camera locations was related to distance between those locations (i.e., to determine if the composition was spatially autocorrelated). Significance levels were determined with permutation tests. Mantel correlograms were implemented in PASSaGE; Mantel tests were implemented in PC-ORD Version 6.12 (McCune & Mefford, 2011).

We used several approaches to analyze differences and similarities in community composition between plots and across years. First, we used a Non-metric Multi-dimensional Scaling ordination (NMS; Bray–Curtis distance measure) to graphically represent similarities (and differences) in species composition between plots and among samples, based on capture rates per species (Clarke & Warwick, 2001; McCune & Grace, 2002). Next, we used analysis-of-similarity (ANOSIM; Bray-Curtis similarity, standardized capture rate; see Clarke & Warwick, 2001) to compare the level of similarity in species composition among annual samples from a given plot (i.e., Puma or Harpia) to the level of similarity across all samples, to determine if plots differed in species composition more than expected by chance. The significance of the ANOSIM test statistic is determined by comparison with values obtained by a Monte Carlo randomization procedure. Ordination was performed with PC-ORD Version 6.12 (McCune & Mefford, 2011); ANOSIM was run with PRIMER Version 6.1.11 (PRIMER-E, 2008).

We used both indirect and direct gradient analyses to examine spatial variation in species composition based on camera location, with data from all years combined. We first used NMS to graphically portray variation in composition among the camera locations, without taking into account effects of environmental variables. Next, we ran detrended correspondence analysis (DCA) to detect gradients in species composition based on rates of capture in cameras. Lengths of the segments for each axis provide an index of the amount of species turnover among sample points (Clark, Palmer & Clark, 1999). Linear models are appropriate and advantageous when the standard deviation of ordination lengths is less than about 3; unimodal models are more appropriate when lengths are greater than 4 (see Clark, Palmer & Clark, 1999). In this case, gradients were all <2.0 so we used redundancy analysis (RDA) to evaluate the relationship between species composition and six environmental features at different camera locations (using 50 and 100-m buffers around each camera location. In RDA biplots, if species and environmental factors are represented by arrows, the cosine of the angle between the arrows approximates the correlation coefficient between the two variables. Arrows in the same direction indicate positive correlation and arrows in the opposite direction indicate negative correlations. Species located farther away from the origin provide more information regarding gradients among samples. DCA and RDA analyses were run on program CANOCO 4 (Ter Braak & Šmilauer, 1998).

We combined data across all years to compare the relative importance of typically terrestrial species (i.e., excluding primates and *Coenodon prehensilis*) by plotting capture rates from both plots combined (*x*-axis) vs capture rates from cameras located along trails at TBS (*y*-axis) following Pitman et al. (2001 see also Blake, 2007; Blake & Loiselle, 2009). Cameras along trails (Fig. 1; Blake et al., 2017) were located approximately 800 to 1,200 m apart. We calculated the slope of the line between plots and trails to test the null hypothesis that capture rates per species did not differ between the two sets of cameras. If true, the slope of the line should not differ from 1.0 (Pitman et al., 2001:2107). Points above a line with slope of 1.0 would indicate greater capture rate on trails; below the line, points would indicate higher rates on plots.

We took the same approach to compare capture rates of species on the two plots combined to capture rates of species at sites located farther from the plots. First, we compared results from cameras located at a site in the eastern part of Yasuní National Park, approximately 90 km ESE of TBS (Arguillin et al., 2010). That site was located in the ITT (Ishpingo, Tambococha, Tiputini) block, an area that has been the focus of questions regarding extraction of oil (Finer et al., 2008; Finer, Moncel & Jenkins, 2010). As with TBS, the area is dominated by *terra firme* forest and had not, at the time of the study, been subjected to substantial disturbance as a result of human activities, although roads used by hunters, tourists, and military personnel were present (Arguillin et al., 2010). Cameras (32 stations) were located 1–2.5 km apart and covered ∼58 km^2^; 603 independent events recorded 28 species in 2015 trap-days. Finally, to examine patterns at a larger scale, we used a similar approach to compare results from the two plots to those from a site in lowland forest in southeastern Peru, approximately 1480 km SSE of TBS, within and adjacent to Los Amigos Conservation Concession (Tobler et al., 2008); part of that study area lies within a logging concession. Cameras were operated in 2005 (24 locations, 2-km grid, ∼50 km^2^) and 2006 (39 locations, 1–2 km grid, ∼50 km^2^). Cameras accumulated 508 images of 21 mammals in 1,440 trap-days in 2005 and 814 images of 27 species in 2,340 trap days in 2006.

Prior to statistical tests, all variables were examined for assumptions of parametric tests and were transformed if necessary. Unless otherwise mentioned, analyses were run with STATISTIX Version 10 (Analytical Software, 2013).

## Results

### General summary

We accumulated a total of 3,533 independent images (images separated by at least 30 min) of mammals, representing a total of 28 species, in 7,189 trap days (Table 1, Table S1). Numbers of images per 100 trap-days was considerably higher on Puma than on Harpia in 2014, 2015 and 2017 but approximately equal in 2016 (Table 1; paired *t*-test, *t* = 2.99, *df* = 3, *P* = 0.058). Despite the lower number of captures, more species were recorded in each year on Harpia than on Puma (paired *t*-test, *t* = 3.29, *df* = 3, *P* = 0.046) but the total across all years was similar on the two plots. Estimated species totals also were higher on Harpia except in 2017; estimated total numbers (all years combined) were similar on the two plots (Table 1).

**Table 1:** Summary of camera-trap surveys. Results of camera-trap surveys on two plots in Tiputini Biodiversity Station, Ecuador, during January–March, 2014–2017. Estimated (Est.) number of species based on Hines (1996). Rates are number of images/100 trap-days.

	Harpia					Puma					Both
	2014	2015	2016	2017	Total	2014	2015	2016	2017	Total	
Trap days	941	964	903	899	3,707	882	842	864	894	3,482	7,189
Species	19	22	19	21	26	15	17	17	20	25	28
Est. species	22	27	23	23	30	16	23	20	23	29	31
Est. species SD	2.4	3.2	2.8	3.2	2.8	1.4	3.5	1.4	2.4	2.8	2.4
Images	338	365	400	386	1,489	538	574	400	532	2,044	3,534
Rate	35.9	37.9	44.3	42.9	40.2	61.0	68.2	46.3	59.5	58.7	49.2

### Individual species

Capture rates across species were similar between plots within a year (2014: *r*_*s*_ = 0.75; 2015: *r*_*s*_ = 0.73; 2016: *r*_*s*_ = 0.85; 2017: *r*_*s*_ = 0.86; years combined: *r*_*s*_ = 0.84; *P* < 0.001 all cases). Capture rates also were similar within plots between years (Harpia: 2014–2015, *r*_*s*_ = 0.83; 2015–2016, *r*_*s*_ = 0.73; 2016–2017, *r*_*s*_ = 0.94; Puma: 2014–2015, *r*_*s*_ = 0.86; 2015–2016, *r*_*s*_ = 0.89; 2016–2017, *r*_*s*_ = 0.80; *P* < 0.001 all cases). Nine species on Harpia had capture rates of at least 1.0/100 trap-days (Table 2), with highest rates recorded for *Pecari tajacu, Mazama americana, Dasyprocta fuliginosa,* and *Cuniculus paca*. Ten species had capture rates ≥1.0/100 trap-days on Puma, with highest rates recorded for *Mazama americana, Tayassu pecari, Dasyprocta fuliginosa, Myoprocta prattii, Pecari tajacu,* and *Tapirus terrestris* (Table 2).

**Table 2:** Capture rates of mammals on two plots. Capture rates (images/100 trap-days) for mammals on two plots in Tiputini Biodiversity Station, Ecuador, during January–March, 2014–2017. Rates are given for each year and for all years combined.

Species	Harpia plot					Puma plot				
	2014	2015	2016	2017	All	2 2014	2015	2016	2017	All
*Didelphis marsupialis*	0.00	0.10	0.00	0.00	0.03	0.00	0.00	0.23	0.00	0.06
*Myrmecophaga tridactyla*	0.32	0.41	0.22	0.56	0.38	0.00	0.00	0.00	0.34	0.09
*Tamandua tetradactyla*	0.00	0.00	0.00	0.11	0.03	0.00	0.12	0.00	0.00	0.03
*Priodontes maximus*	0.43	0.31	0.89	0.44	0.51	0.34	0.36	0.58	0.34	0.40
*Dasypus novemcinctus*	1.81	2.59	4.10	3.23	2.91	6.80	1.43	3.59	4.36	4.08
*Saguinus tripartitus*	0.00	0.10	0.00	0.00	0.03	0.00	0.00	0.00	0.00	0.00
*Cebus albifrons*	0.00	0.00	0.00	0.33	0.08	0.00	0.00	0.00	0.00	0.00
*Ateles belzebuth*	0.00	0.00	0.00	0.00	0.00	0.00	0.12	0.00	0.00	0.03
*Atelocynus microtis*	0.53	1.35	0.11	0.22	0.57	0.00	0.00	0.23	0.00	0.06
*Procyon cancrivorus*	0.11	0.00	0.00	0.00	0.03	0.00	0.12	0.12	0.22	0.11
*Nasua nasua*	0.11	0.10	0.22	0.22	0.16	0.68	0.12	0.23	0.22	0.32
*Eira barbara*	0.00	0.00	0.11	0.11	0.05	0.34	0.00	0.00	0.11	0.11
*Leopardus pardalis*	0.43	0.83	0.33	1.00	0.65	2.49	0.95	0.58	2.13	1.55
*Leopardus weidii*	0.00	0.41	0.00	0.00	0.11	0.00	0.12	0.00	0.00	0.03
*Puma concolor*	0.74	0.10	4.98	1.33	1.75	1.02	0.12	2.89	1.01	1.26
*Puma yagouaroundi*	0.11	0.10	0.11	0.00	0.08	0.00	0.00	0.00	0.00	0.00
*Panthera onca*	0.32	0.62	0.66	0.44	0.51	0.23	0.71	1.39	0.22	0.63
*Tapirus terrestris*	1.17	0.83	1.33	1.33	1.16	5.56	8.43	3.94	5.93	5.94
*Pecari tajacu*	13.28	13.69	10.41	15.46	13.22	9.52	9.26	5.09	5.93	7.44
*Tayassu pecari*	1.70	0.62	1.44	3.00	1.67	6.92	8.31	6.71	8.84	7.70
*Mazama americana*	2.44	5.71	8.53	7.45	5.99	6.58	14.01	7.18	10.29	9.48
*Mazama nemorivaga*	0.74	0.21	1.00	0.11	0.51	0.00	0.00	0.35	0.11	0.11
*Sciurus igniventris*	0.85	0.21	0.11	0.11	0.32	0.00	0.00	0.00	0.45	0.11
*Coendou prehensilis*	0.00	0.00	0.00	0.00	0.00	0.00	0.00	0.00	0.11	0.03
*Cuniculus paca*	5.42	3.11	3.10	3.45	3.78	1.70	3.21	3.01	4.14	3.02
*Dasyprocta fuliginosa*	4.04	4.15	4.10	2.89	3.80	7.14	6.29	1.62	7.49	5.66
*Myoprocta prattii*	1.38	2.18	2.55	1.00	1.78	11.56	14.49	8.56	6.94	10.34
*Proechimys* sp*.*	0.00	0.21	0.00	0.11	0.08	0.11	0.00	0.00	0.34	0.11

**Figure 2:**
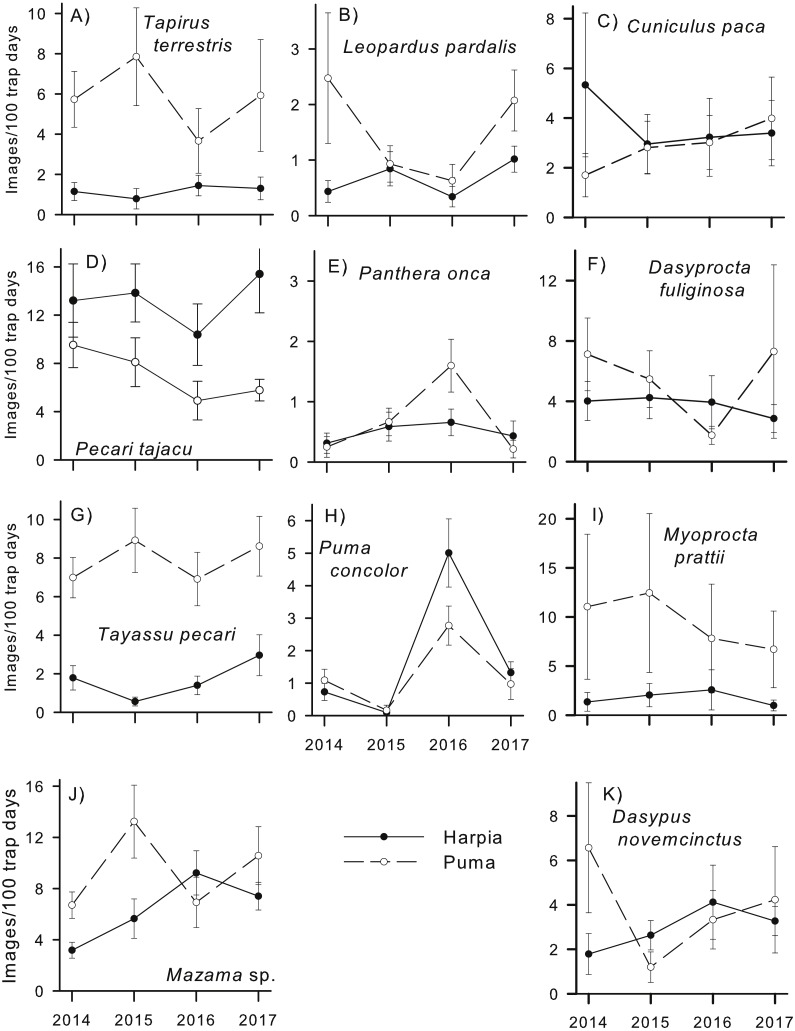
Capture rates of common mammals. Capture rates (mean number of images/100 trap-days ± SE, calculated from capture rates at individual camera locations) by year for common species on two plots (Harpia, Puma) at Tiputini Biodiversity Station, Ecuador. (A) *Tapirus terrestris*; (B) *Leopardus pardalis*; (C) *Cuniculus paca*; (D) *Pecari tajacu*; (E) *Panthera onca*; (F) *Dasyprocta fuliginosa*; (G) *Tayassu pecari*; (H) *Puma concolor*; (I) *Myoprocta pratti*; (J) *Mazama* spp.; (K) *Dasypus novemcinctus*.

Some species exhibited large and consistent differences in capture rates between plots (Fig. 2). *Tapirus terrestris, Tayassu pecari*, and *Myoprocta prattii* were more common on Puma during each year whereas *Pecari tajacu* was the only common species with consistently higher capture rates on Harpia. Others, such as *Leopardus pardalis, Mazama* sp. and *Dasyprocta fuliginosa*, differed in capture rates between plots but direction and extent of the difference varied across years. Still other relatively common species, such as *Cuniculus paca, Dasypus novemcinctus, Puma concolor* and *Panthera onca*, showed little difference between plots in most years. Similarly, species differed in extent of variation in capture rates among cameras within a given year and plot. Capture rates of *Myoprocta pratti*, for example, were highly variable (large SEs) among cameras on Puma (Fig. 2).

**Table 3:** Spatial analyses of mammals based on data from camera traps. Spatial indices for species represented by at least 10 images on a given plot. Index of Dispersion (ID) and associated *P*-values indicate whether images were significantly clumped (i.e., significantly > 1.0) or not. Also shown are *P*-values for Moran’s I (MI), which evaluates whether captures at camera locations were spatially autocorrelated; significance values are given without correction for multiple comparisons.

Species	Harpia Plot			Puma Plot		
	ID	*P* =	MI: *P* =	ID	*P* =	MI: *P* =
*Myrmecophaga tridactyla*	0.74	0.74	0.33			
*Priodontes maximus*	1.15	0.31	0.16	3.03	0.001	1.00
*Dasypus novemcinctus*	5.68	0.001	1.00	17.52	0.001	0.11
*Atelocynus microtis*	4.44	0.001	1.00			
*Nasua nasua*				1.69	0.05	
*Leopardus pardalis*	1.24	0.23	0.28	3.55	0.001	0.62
*Puma concolor*	1.92	0.017	0.10	1.62	0.06	1.00
*Panthera onca*	1.26	0.22	1.00	1.15	0.30	1.00
*Tapirus terrestris*	2.32	0.003	1.00	21.30	0.001	1.00
*Pecari tajacu*	9.00	0.001	0.46	9.30	0.001	0.56
*Tayassu pecari*	3.20	0.001	1.00	3.12	0.001	0.71
*Mazama americana*	6.72	0.001	0.04	7.81	0.001	0.92
*Mazama nemorivaga*	1.15	0.31	1.00			
*Sciurus igniventris*	2.58	0.001	1.00			
*Cuniculus paca*	9.65	0.001	1.00	10.04	0.001	0.28
*Dasyprocta fuliginosa*	8.26	0.001	1.00	31.55	0.001	1.00
*Myoprocta prattii*	13.80	0.001	1.00	152.7	0.001	1.00

Amount of variation in capture rates across years differed among species. Some, such as *Myoprocta prattii, Tayassu pecari, Dasyprocta fuliginosa, Tapirus terrestris*, and *Cuniculus paca* showed very little change from one year to the next, at least on one plot (e.g., *Dasyprocta fuliginosa* and *Tapirus terrestris* showed more variation across years on Puma than on Harpia). In contrast, other species, such as *Puma concolor* and *Mazama americana* showed substantial variation among years. In the case of *P. concolor*, the increase in capture rates from 2014–2015 to 2016 followed by a decrease to 2017 was especially notable, particularly as it occurred on both plots. In contrast*, Leopardus pardalis* showed a large decrease in capture rates on Puma from 2014 to 2015 with a large increase from 2016 to 2017.

Of 17 species represented by at least 10 images on at least one plot (all years combined; 16 species on Harpia, 13 on Puma), 11 demonstrated a clumped distribution pattern (Index of Dispersion, ID, significantly different from random) on each plot (Table 3). *Panthera onca* was the only species that did not show a clumped distribution pattern on either plot. *Priodontes maximus* and *Leopardus pardalis* did not show a clumped pattern on Harpia but did on Puma. Indices of dispersion were similar on both plots for some species (e.g., *Puma concolor, Pecari tajacu, Tayassu pecari, Cuniculus paca*) but differed strongly for others (e.g., *Dasypus novemcinctus, Tapirus terrestris, Dasyprocta fuliginosa, Myoprocta prattii*); all of the latter species had higher indices (stronger clumping pattern) on Puma than on Harpia (Table 3).

Despite the fact that a number of species gave evidence of a clumped distribution pattern, there was little evidence to suggest that numbers of images in particular cameras were correlated with numbers of images in other cameras—i.e., there was little to no spatial autocorrelation in numbers of images (based on Bonferroni corrected significance values; Table 3). A significant autocorrelation was noted for *Mazama americana* on Harpia but this reflected autocorrelation with distant camera locations (∼300–400 m away), suggesting that the autocorrelation was not ecologically meaningful (and not significant after correction for multiple comparisons). Thus, overall, there was little evidence to suggest that captures of a given species at one camera location were related to or influenced by captures at other cameras, even for species, such as most felids, that regularly travel along transects. Mantel correlograms showed no significant autocorrelations in species composition at any distance category. Similarly, results of Mantel tests indicated no significant correlation between composition of captures at camera locations and distance between cameras (Harpia—standardized Mantel statistic, *r* = 0.14, *P* = 0.22; Puma, *r* = 0.12, *P* = 0.32).

**Figure 3:**
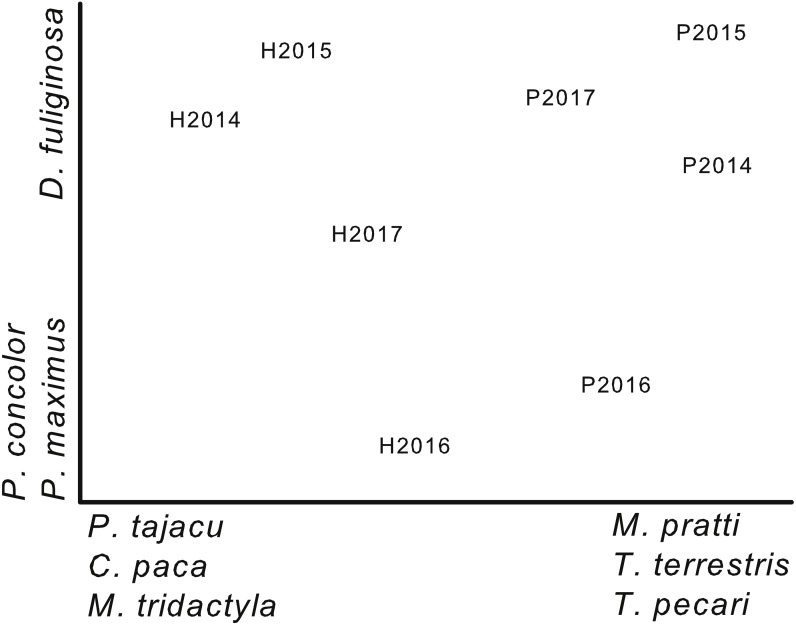
Ordination of plots by year based on capture rates. Non-metric multi-dimensional scaling ordination based on capture rates of 17 terrestrial mammals during 4 years (2014–2017) on two plots (Harpia—H; Puma—P) at Tiputini Biodiversity Station, Ecuador. Species most highly correlated with axes are shown. Final stress value was 0.06.

### Community composition

A NMS ordination based on capture rates of 17 terrestrial species (species represented by at least 10 captures; cameras combined on each plot for each year) separated samples from the two plots (Fig. 3). From left to right, the first axis of the ordination reflected a gradient of decreasing importance of species such as *Pecari tajacu*, which was more important on Harpia, and increasing importance of others, such as *Tayassu pecari*, which was more important on Puma. This separation reflects the differences in capture rates between plots (Table 2). In contrast, the second axis primarily reflected differences in capture rates among years, rather than between plots. *Puma concolor*, for example, was more commonly encountered in 2016 on both plots than in other years. Results of an ANOSIM indicated that samples from each plot were, overall, more similar to each other than to samples from the other plot (global *R* = 0.9, *P* = 0.029).

### Spatial variation among camera locations

**Figure 4:**
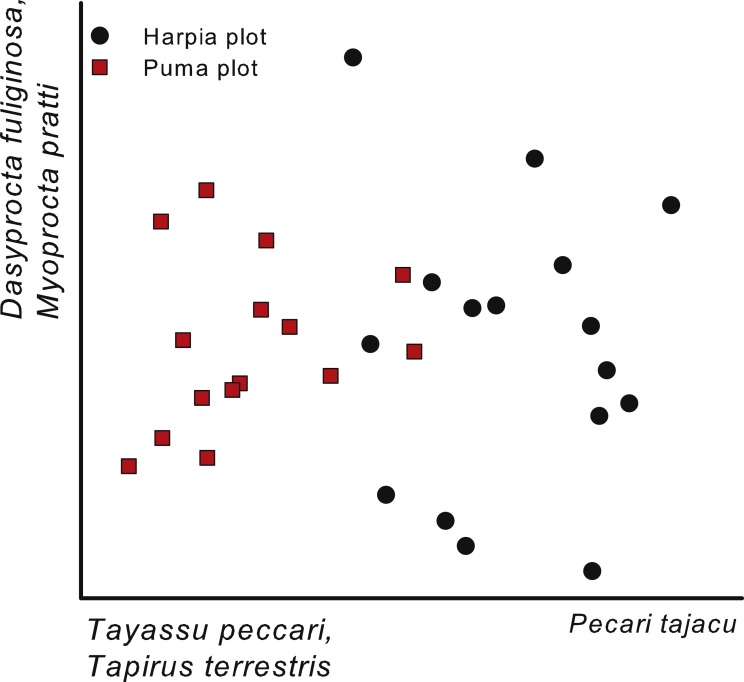
Ordination of camera trap locations. Non-metric multi-dimensional scaling ordination of camera locations based on images of 17 common species, combined across four years (2014–2017) on two plots at Tiputini Biodiversity Station, Ecuador. Species most highly correlated with axes are shown. Final stress value was 0.11.

A NMS based on capture rates (ln-transformed) from individual camera locations (all years combined) also indicated a separation between the two plots (Fig. 4). As with the comparison across years (Fig. 3; cameras combined within a year), the first axis of the ordination reflected differences in importance of peccaries, with *Tayassu pecari* more important on Puma and *Pecari tajacu* on Harpia. In addition, the gradient also reflected greater importance of *Tapirus terrestris* on Puma. The second axis largely reflected differences in importance of *Dasyprocta fuliginosa* and *Myoprocta pratti* and did not indicate a separation between plots, but rather differences among cameras irrespective of plot (Fig. 4); no species showed a strong negative correlation with the second axis.

**Table 4:** Redundancy analyses of camera-trap images. Redundancy analyses relating camera-trap rates of 17 species of mammals to environmental variables measured within 50 and 100 m around each camera trap location on two plots (Harpia, Puma) at Tiputini Biodiversity Station, Ecuador. Results are based on data combined for both plots.

	Axes			
	1	2	3	4
50 m buffer				
Eigenvalues	0.105	0.090	0.057	0.027
Species-environmental correlations	0.788	0.816	0.766	0.573
Cumulative percentage variance of species data	10.5	19.5	25.2	27.9
Cumulative percent. variance of species-environmental relation	34.0	63.4	81.8	90.7
Test of significance of first axis: *F* = 2.92, *P* = 0.060				
Test of significance of all canonical axes: *F* = 1.85, *P* = 0.002				
100 m buffer				
Eigenvalues	0.142	0.074	0.051	0.028
Species-environmental correlations	0.880	0.774	0.798	0.591
Cumulative percentage variance of species data	14.2	21.6	26.8	29.5
Cumulative percent. variance of species-environmental relation	44.4	67.6	83.6	92.2
Test of significance of first axis: *F* = 4.14, *P* = 0.004				
Test of significance of all canonical axes: *F* = 1.96, *P* = 0.002				

**Figure 5:**
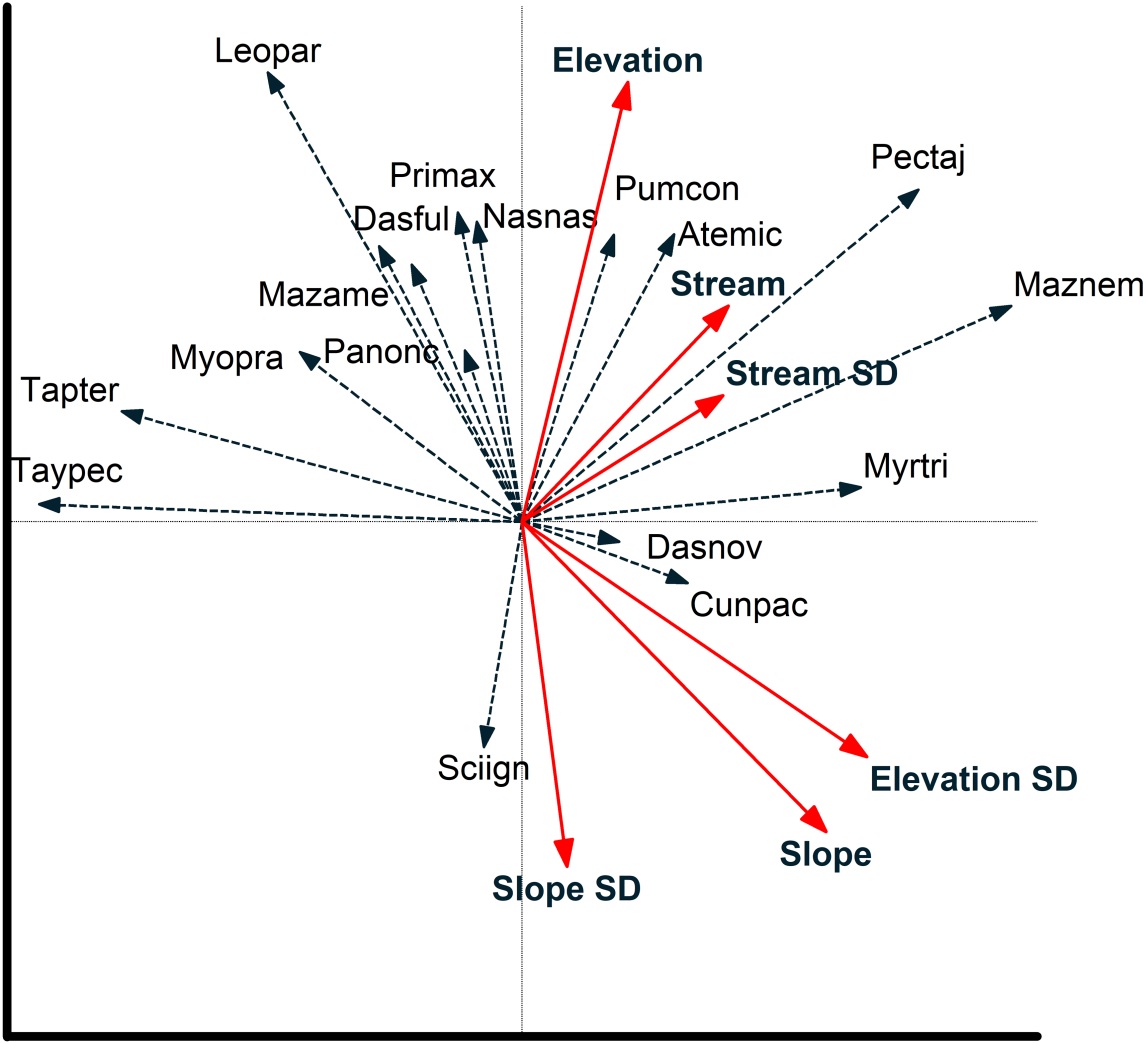
Species environmental relationships based on redundancy analysis. Relationship between environmental variables and individual species (species are coded by first three letters of genus and first three letters of species; see Table 2) from a redundancy analysis based on images of 17 mammal species recorded at cameras on two plots at Tiputini Biodiversity Station, Ecuador. Images were combined across four years (2014–2017). Environmental variables were calculated based on a 50-m radius around each camera location. See text for details on interpreting biplots.

**Figure 6:**
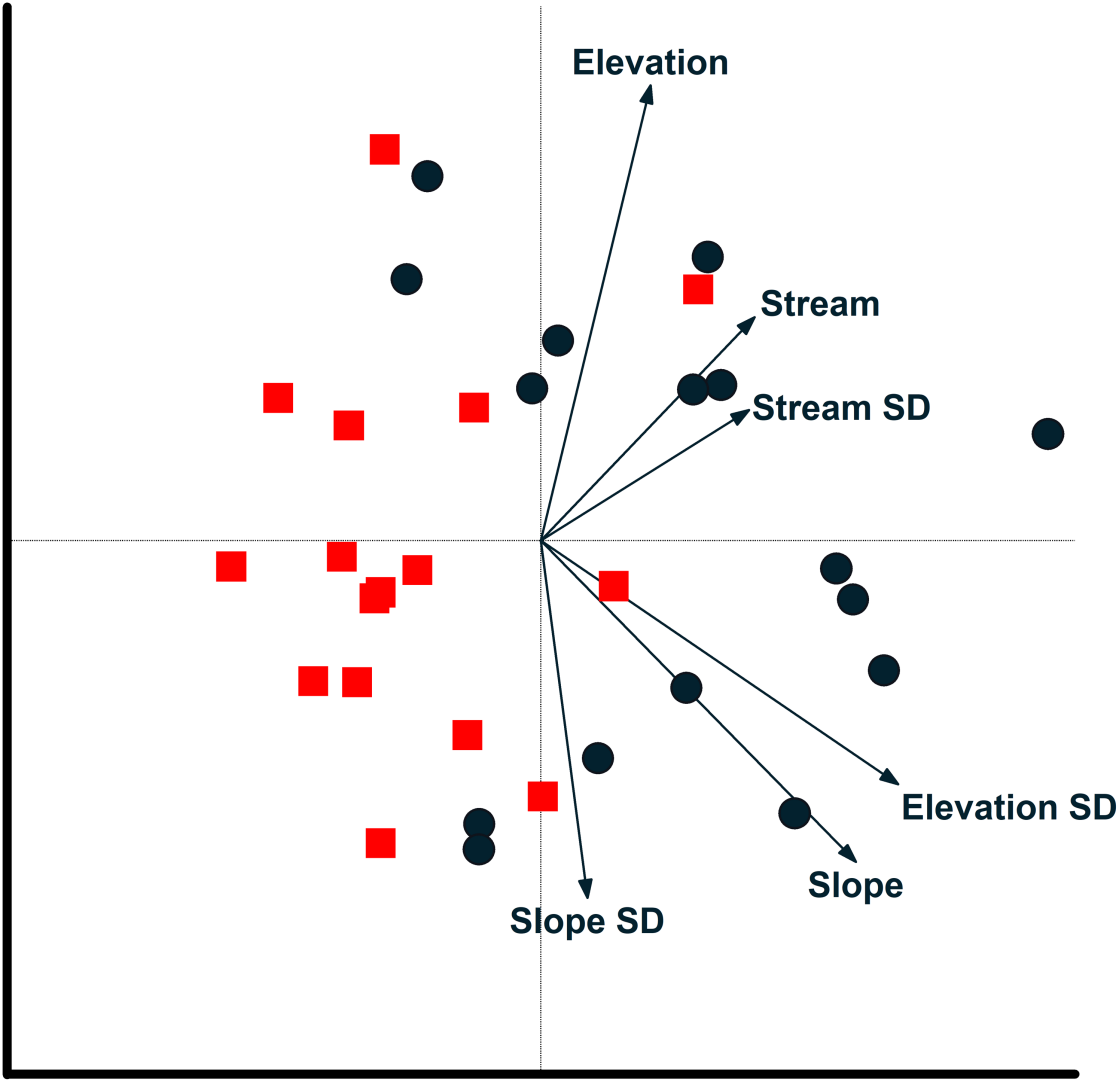
Camera locations in relation to environmental factors based on redundancy analysis. Relationship between camera locations and environmental variables from a redundancy analysis based on images of 17 mammal species recorded at cameras on two plots at Tiputini Biodiversity Station, Ecuador. Images were combined across four years (2014–2017). Environmental variables were calculated based on a 50-m radius around each camera location. See text for details on interpreting biplots. Harpia plot—circles; Puma plot—squares.

### Influence of environmental variation

Mean elevation did not differ among camera locations on the two plots at either the 50 or 100-m buffer scale (Table S2) but elevation was more variable on Harpia at both scales (i.e., significant differences in standard deviation values), reflecting the hillier terrain on Harpia. Similarly, slope and distance to streams were greater on Harpia, although significant only at the 100-m scale. These differences reflect the fact that the Puma plot tends to be flatter and with more permanent streams in comparison with Harpia.

Redundancy analysis based on environmental features within 50-m buffer around cameras explained a small but significant amount of variation in the distribution of species (Table 4). The first two axes accounted for almost 20% of variation in species distribution patterns, with slope, elevation, SD of slope, and SD of distance to stream all significant in a stepwise analysis. Thus, species such as *Tayassu pecari* and *Tapirus terrestris* were negatively associated with steeper slopes and greater variation in elevation (Fig. 5). Similarly, *Leopardus pardalis* was strongly negatively associated with slope and variation in slope and elevation. In contrast, *Puma concolor* and *Atelocynus microtis* were positively associated with elevation whereas *Pecari tajacu* tended to occur in areas farther from streams. In general, cameras on Puma separate from those on Harpia along the first axis; cameras on Puma tend to be in flatter areas and closer to streams, although this is not true for all camera locations on the plot (Fig. 6).

Environmental features explained slightly more variation in species distribution patterns when based on a 100-m buffer around camera locations (Table 4). Slope, SD of slope, and elevation all were significant in a stepwise analysis. Relationships between species and environmental features tended to be similar to those based on 50-m buffer (Fig. S1). *Tayassu pecari* and *Tapirus terrestris* were even more strongly negatively associated with variation in elevation, *Pecari tajacu* was more strongly associated with distances to streams, but *Atelocynus microtis*, for example, was not strongly associated with any variable. Cameras on Puma showed less variation in elevation than did cameras on Harpia and tended to be in areas with lower slopes (Fig. S1).

### Comparison with other sites

**Figure 7:**
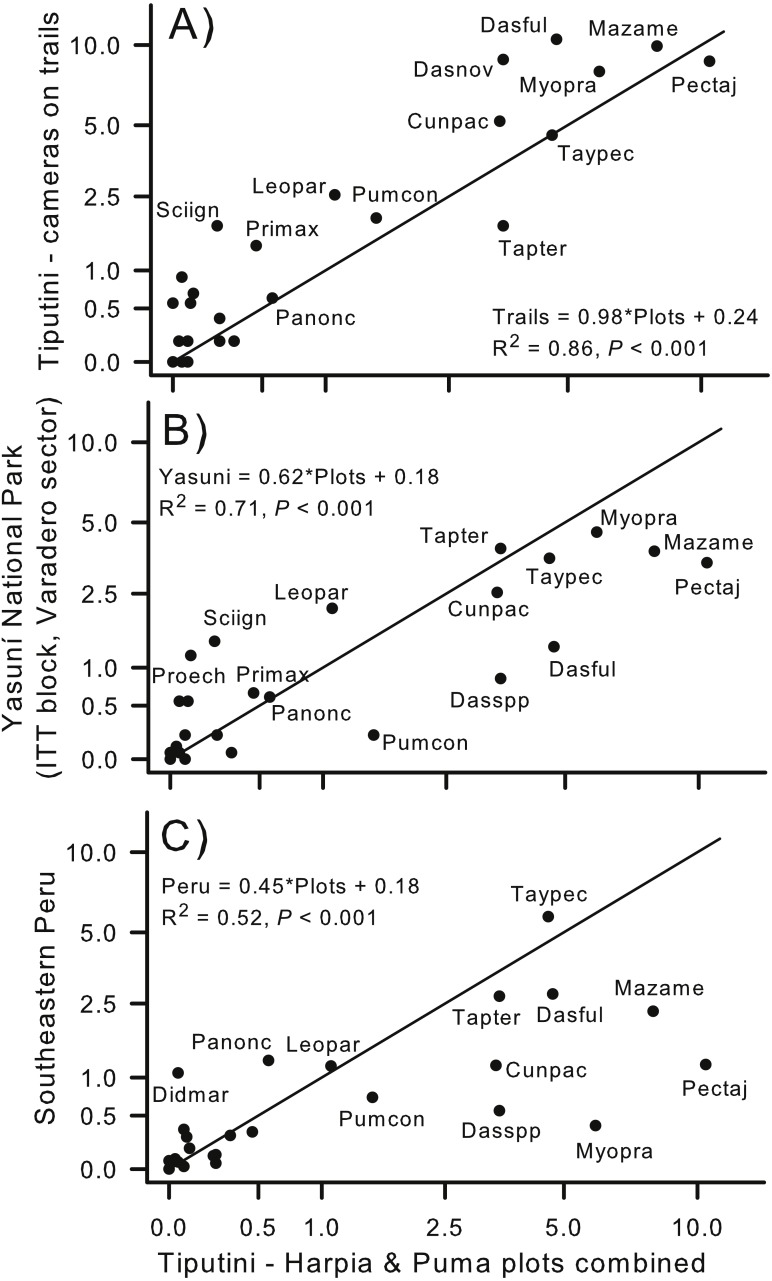
Comparison of capture rates at sites in Ecuador and Peru. Comparison of capture rates of mammals on two plots (combined) at Tiputini Biodiversity Station (TBS), Ecuador, with capture rates of mammals from cameras located (A) along trails at TBS (e.g., see Blake et al., 2017); (B) located at a site within Yasuni National Park (Arguillin et al., 2010; ∼90 km from TBS); and (C) cameras located at a site in southeastern Peru (Tobler et al., 2008; ∼1,480 km from TBS). Solid line indicates a 1:1 slope (i.e., equal rates in both areas being compared). Regression equations are shown for each comparison.

Capture rates of species on the two plots combined (all years combined) were similar to capture rates from cameras located on trails within the boundaries of TBS (Fig. 1; *r*_*s*_ = 0.84; Fig. 7A). Capture rate of *Tapirus terrestris* was somewhat higher on the plots than on the trails whereas *Sciurus igniventris*, *Leopardus pardalis*, and *Dasypus novemcinctus* were somewhat more commonly encountered on trails. In contrast, capture rates were, overall, much higher on the two combined plots than at a different site within Yasuní National Park (ITT block, Varadero sector; Arguillin et al., 2010). Most species had higher capture rates on the two plots, as indicated by points lying below the line that indicates a 1:1 relationship (Fig. 7B). The lower slope was strongly influenced by species such as *Puma concolor*, *Dasypus* spp., *Dasyprocta fuliginosa*, and *Mazama americana*. Despite these differences, relative (rank) importances of species were similar in both sites (*r*_*s*_ = 0.81). Similarly, the slope of the relationship was much less than 1.0 when results from TBS were compared to a site in southeastern Peru (Tobler et al., 2008). For example, *Pecari tajacu*, *Mazama americana*, and *Myoprocta pratti* all were captured much more frequently on the TBS plots whereas *Panthera onca* and *Didelphis marsupialis* were more commonly captured at the Peru site (Fig. 7C). Capture rates were, nonetheless, highly correlated (*r*_*s*_ = 0.79) indicating that relative importance of species was similar in lowland forest of eastern Ecuador and in southeastern Peru.

## Discussion

Terrestrial mammal assemblages on two plots in relatively close proximity (<2 km apart) in lowland forest of eastern Ecuador were similar in terms of overall species richness and composition; most species were found on both plots. Yet, despite the close proximity, the assemblages differed in a number of ways. Although total species numbers were similar on both plots when all four years were combined, on an annual basis species richness was higher on one plot than on the other. In contrast, the numbers of images (capture rates) were higher on the other plot, throughout the course of the study. Capture rates of individual species also differed between plots with some species encountered more frequently on one plot or the other. As a consequence, annual samples from one plot were more similar to each other than to samples from the other plot. Thus, even though both sample plots have been influenced by similar historical and regional processes, small-scale differences in habitat and species distribution patterns were evident.

Capture rates were considerably higher on Puma than on Harpia with all years combined and in three of four years separately; rates were most similar in 2016. The higher overall capture rate on Puma largely reflected higher captures of species such as *Myoprocta pratti*, *Tayassu pecari*, and *Tapirus terrestris*; *Pecari tajacu* was the only species that had a consistently much higher capture rate on Harpia. Although both plots were in *terra firme* forest, there nonetheless were some distinct differences in habitat conditions that likely influenced the distribution patterns of species. Puma, for example, is flatter overall than Harpia (although the total elevational range is about the same), shows less variation in elevation and slope and has more permanent streams. These factors were important in accounting for variation in distribution patterns of a number of species (based on RDA with 50 and 100-m buffers around cameras). *Tapirus terrestris* and *Tayassu pecari*, for example, were strongly negatively associated with variation in elevation and degree of slope; both species are encountered much more often on Puma although both also occur on Harpia.

Although the environmental variables included in this analysis accounted for a significant amount of variation in species distribution patterns, most variation remained unaccounted for indicating that additional factors likely influenced distribution patterns of species. Differences in availability (temporal and spatial variation) of resources (e.g., *Ficus* fruits), for example, likely help explain the greater activity of species such *Myoprocta pratti* and *Tapirus terrestris* on the Puma plot; individuals of both species were frequently photographed eating fruit. Given that home ranges of most species extend beyond the area sampled by an individual camera, or even beyond the entire grid of cameras, occurrence of species at a given camera location may be influenced by factors operating at larger spatial scales. Distribution and abundance of prey, for example, might be expected to influence the occurrence and activity of large felids (Harmsen et al., 2011; Foster et al., 2013). Occurrence of specific habitat types also might influence distribution patterns of species. Although none of the cameras used in the current study were placed at mineral licks, such sites are frequented by many species encountered in this study (Blake et al., 2011 and references therein). Consequently, the location of licks might influence movement patterns of some species which, in turn, might affect the likelihood of encountering cameras.

Capture frequencies of species were highly correlated both between plots and within a plot between years despite differences in topographic conditions that apparently influenced distribution patterns of individual species. A similar result was reported by Tobler et al. (2008), where the correlation between two-month surveys conducted a year apart was high (*r*_*s*_ = 0.88; Tobler et al., 2008: 173). They suggested that the high correlation meant that capture rates might be species specific. Nonetheless, other factors clearly influence capture frequencies in a given year as many species showed substantial variation in numbers of images across years.

Images of most species were not evenly distributed across cameras within a plot but rather showed a clumped distribution pattern. Such a pattern suggests spatial variation in habitat conditions on each plot, as shown by the ordination of camera locations relative to topographical variables. The lack of autocorrelation in captures among cameras suggests that the clumped pattern of images likely reflects relatively small-scale variation in habitat conditions (e.g., topography, distance to streams) or resource availability. The lack of autocorrelation also suggests that captures at adjacent cameras were relatively independent for many, but not all, species. *Panthera onca* was the only species that did not show a clumped distribution on either plot despite the fact that prey species (e.g., deer, peccaries) did. Jaguars move over large ranges frequently along trails and may be less affected by relatively small-scale variation in topography or other habitat conditions.

Based on range maps and descriptions in Emmons & Feer (1997), ∼31 species of medium to large-sized (i.e., >300–500 g) terrestrial (i.e., not primarily arboreal, non-aquatic) mammals might be expected to occur in or near TBS, very close to the estimated totals of 30 and 29 species on Harpia and Puma, respectively, and equal to the estimated total with both plots combined. Of these 31 expected species, 24 were recorded by camera traps on the two plots combined. (Twenty-eight species were recorded on both plots combined but that total included three primates and a porcupine, all of which are primarily arboreal.) Species that were not recorded included three that have been detected in other cameras located on trails within the station boundary: *Metachirus nudicaudatus* (one image), *Speothos venaticus* (two images), and *Sylvilagus brasilianus* (37 images; Blake et al., 2012; Blake et al., 2017). *Speothos* also has been seen on one or two occasions. Although *Sylvilagus* has been recorded more frequently, images are from few locations, so this species is apparently not widespread across the station. According to Emmons & Feer (1997), *Sylvilagus* prefers areas near swamps, river edges, and other open areas, habitats not well represented on the two plots. Other species which might occur in the area but that have not been recorded by cameras, either on the plots or along trails, include *Philander andersoni* (which prefers vine tangles near water; Emmons & Feer, 1997), *Galictis vittata* (also found near rivers and streams), *Cabassous unicinctus* (“apparently uncommon or rare” everywhere; Emmons & Feer, 1997), and *Dasypus kappleri*. The last species may occur at TBS but is difficult to distinguish from *D. novemcinctus* in images.

Ignoring the three species that have never been recorded by cameras at Tiputini Biodiversity Station (i.e., *Philander andersoni*, *Galictis vittata*, *Cabassous unicinctus*), we might expect that ∼28 species could be recorded by cameras on the two plots. Thus, the 24 species that were recorded on the two plots (all years combined) is ∼86% of the possible total, suggesting that the mammal fauna was well sampled. On the other hand, in any given year, the percentage of this total ranged from 68 to 75% on Harpia but only 53 to 68% on Puma. This is in contrast to a site in lowland forest in southeastern Peru, where Tobler et al. (2008) recorded 75% and 86% of 28 species reported from the study area in only two months in each of two years (1,440 and 2,340 trap days/year in the two years sampled). As at TBS, species that were not recorded in their survey were extremely rare, including several not recorded in the current study at TBS (*Galictis vittata, Cabassous unicinctus, Speothos venaticus*). Cameras at TBS covered a much smaller sampling area than in the Peru study (∼50 km^2^) which may help explain why a smaller percentage of species were detected in a given year at TBS. A larger sampling area would presumably cover a broader range of habitats, increasing the probability of detecting more species. If the goal of a study is to acquire the most complete representation of likely species within a short period, then cameras spread out over a larger area may be more efficient. Such larger-scale surveys would not, however, provide as much detailed information on the composition of mammals within a given area and how that might change over time.

Despite apparent differences in capture rates, across all spatial scales, the relative importance of different species of mammals recorded in camera traps in lowland Amazonian forests was very similar, as was the total number of species recorded in the different studies. This was true even given differences in trap spacing, levels of disturbance, and length of study. Capture rates and species composition of mammals on the two plots included in this study were very similar to those recorded in camera traps set along trails within the boundaries of TBS. This similarity in composition is perhaps not surprising as all cameras were in relatively close proximity (TBS encompasses ∼650 ha) and located in *terra firme* forest. Cameras on trails were farther apart (∼800–1,200 m) than those on the two plots and closer to the spacing seen in many other camera-trap studies (Burton et al., 2015). The high correlation in capture rates between plots and trails likely reflects the fact that plots and trails are and have been influenced by similar environmental conditions and have experienced similar, low levels of human disturbance (e.g., no hunting pressure). Despite the overall similarity, some species did exhibit differences in frequency of capture between plots and between plots and trails. *Tapirus terrestris*, for example, was encountered more often on plots (particularly Puma) than along trails, which may reflect the fact that cameras on Puma were typically in flatter locations and closer to streams.

Differences in assemblage composition were somewhat more pronounced when results of the current study were compared to results from a site in *terra firme* forest in Yasuní National Park (Arguillin et al., 2010), approximately 90 km away. That site does experience somewhat greater levels of human disturbance (some of the camera locations were along roads used by a variety of hunters, tourists, and military vehicles; Arguillin et al., 2010) although levels of human activity were still low. Most species were more frequently recorded on cameras at TBS, with the notable exceptions of *Leopardus pardalis* and *Sciurus igniventris*. Cameras at the Yasuní sites were located along roads and trails, which may help explain the greater frequency of *Leopardus* records. *Panthera onca* was, however, equally common in both locations and *Puma concolor* was more frequent at TBS; both of these species also frequently use trails and roads as travel routes. Despite these differences, overall capture rates were still highly correlated between Tiputini and Yasuní. Finally, at an even greater spatial scale, differences in capture frequencies were even more pronounced between TBS and a site in southeastern Peru (Tobler et al., 2008) approximately 1,480 km away), although still highly correlated. Species such as *Pecari tajacu*, *Myoprocta pratti*, and *Mazama americana* were encountered much more often in TBS. In contrast, capture rates of *Tayassu pecari* were approximately equal in the two sites and *Panthera onca* was more common at the Peru site. It is important to consider, however, that many factors may influence the rate at which species are recorded by cameras (see comments by Tobler et al., 2008) and these apparent differences in capture rates do not, necessarily, reflect actual differences in abundance among sites. Rather, they reflect differences in levels of activity in locations sampled by specific cameras, which may be influenced by a variety of factors unrelated to actual abundance.

## Conclusions

Results from this study indicate that small-scale differences in environmental conditions can influence the distribution and composition of terrestrial mammal assemblages. Distribution and capture rates of many species exhibited different patterns of temporal and spatial variation despite the fact that both sample plots certainly been influenced by similar historical and regional processes. Images of most species showed a clumped distribution pattern on each plot, likely reflecting, at least in part, spatial variation in habitat conditions on each plot. Further, the lack of spatial autorcorrelation in distribution of images further suggests that many species were responding to differences in factors that operated at the level of camera locations. Nonetheless, environmental variables included in this analysis only accounted for a relatively small, but significant, amount of variation in species distribution patterns. Consequently, other factors, perhaps operating at larger spatial scales, accounted for a greater amount of variation. Although individual species showed considerable variation in capture rates when results from this study were compared to those at other sites, across all spatial scales, the relative importance of mammals recorded in camera traps in lowland Amazonian forests was very similar, as was the total number of species recorded in the different studies.

##  Supplemental Information

10.7717/peerj.4241/supp-1 Supplemental Information 1 Image data, camera coordinates, and environmental data Images are identified by species, plot, camera location, and year. A second sheet provides plot coordinates for the camera locations on each plot. Additional sheets provide environmental data used in redundancy analyses.

10.7717/peerj.4241/supp-2 Figure S1 Redundancy analysis based on environmental variables within 100 m of camera traps Redundancy analysis based on images of 17 species of mammals recorded at cameras on two plots at Tiputini Biodiversity Station, Ecuador. Images were combined across four years (2014–2017). Environmental variables were calculated based on a 100-m radius around each camera location. (A) Relationship between environmental variables and individual species (species are coded by first three letters of genus and first three letters of species; see Table 2). (B) Relationship between camera locations and environmental variables. See text for details on interpreting biplots. Harpia plot—circles; Puma plot—squares.

10.7717/peerj.4241/supp-3 Table S1 Number of images by species and year Number of independent records (at least 30-min separation) of mammals from camera traps located on two study plots in Tiputini Biodiversity Station, Ecuador, during January–March 2014–2017 (14, 15, 16, 17).

10.7717/peerj.4241/supp-4 Table S2 Variables used to characterize environmental conditions around camera traps Values (mean, SE) of variables used to characterize environmental conditions within either 50 m or 100 m radius of camera locations on two plots in lowland forest at Tiputini Biodiversity Station, Ecuador. Values are given for both plots combined and for each plot separately. Comparisons between plots were based on two-sample *t*-tests.

## Additional Information and Declarations

### Field Study Permissions

The following information was supplied relating to field study approvals (i.e., approving body and any reference numbers): Work at Tiputini Biodiversity Station was approved by the Ministerio del Ambiente, Ecuador. Approval number: No. 0005-FAU-MAE-DPOPNY.
