# Supplementary figures and images for: Annual and spatial variation in composition and activity of terrestrial mammals on two replicate plots in lowland forest of eastern Ecuador
Source: PeerJ. 2018 Jan 9;6:e4241. doi: 10.7717/peerj.4241 (PMC5765811; doi:10.7717/peerj.4241)

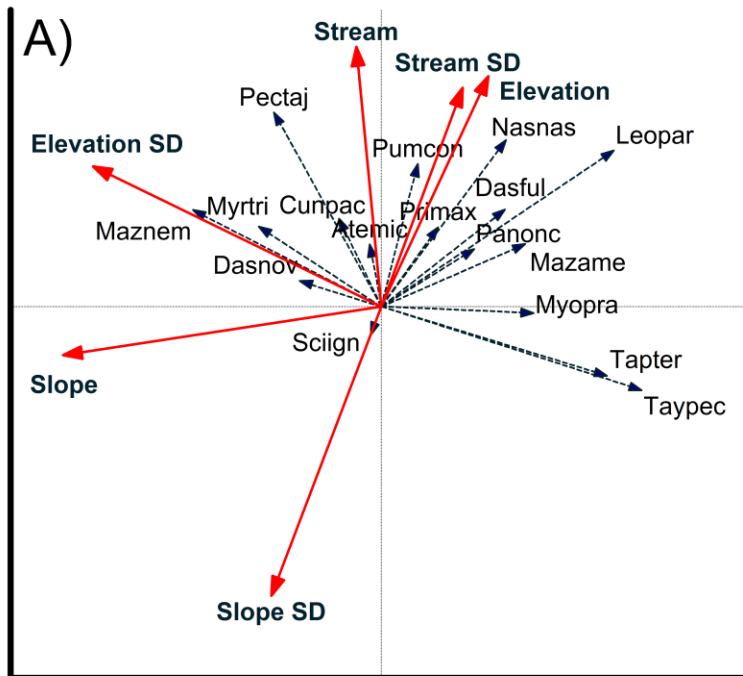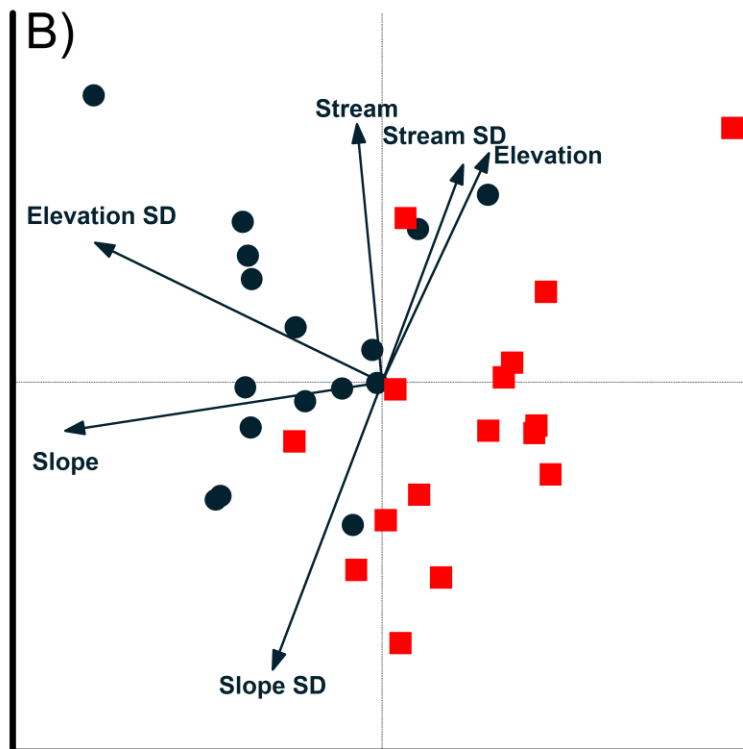

Supplement: Figure S1 — Redundancy analysis based on images of 17 species of mammals recorded at cameras on two plots at Tiputini Biodiversity Station, Ecuador. Images were combined across four years (2014–2017). Environmental variables were calculated based on a 100-m radius around each camera location. (A) Relationship between environmental variables and individual species (species are coded by first three letters of genus and first three letters of species; see Table 2). (B) Relationship between camera locations and environmental variables. See text for details on interpreting biplots. Harpia plot—circles; Puma plot—squares. [file peerj-06-4241-s002.pdf]
