# Supplementary material for: Annual and spatial variation in composition and activity of terrestrial mammals on two replicate plots in lowland forest of eastern Ecuador
Source: PeerJ. 2018 Jan 9;6:e4241. doi: 10.7717/peerj.4241 (PMC5765811; doi:10.7717/peerj.4241)
Supplement: Table S1 — Number of independent records (at least 30-min separation) of mammals from camera traps located on two study plots in Tiputini Biodiversity Station, Ecuador, during January–March 2014–2017 (14, 15, 16, 17). [file peerj-06-4241-s003.docx]

| Family/Scientific name | Common name | Harpia Plot | | | | Puma Plot | | | |
| --- | --- | --- | --- | --- | --- | --- | --- | --- | --- |
|  |  | 2014 | 2015 | 2016 | 2017 | 2014 | 2015 | 2016 | 2017 |
| Didelphidae |  |  |  |  |  |  |  |  |  |
| *Didelphis marsupialis* | common opossum |  | 1 |  |  |  |  | 2 |  |
| Myrmecophagidae |  |  |  |  |  |  |  |  |  |
| *Myrmecophaga tridactyla* | giant anteater | 3 | 4 | 2 | 5 |  |  |  | 3 |
| *Tamandua tetradactyla* | southern tamandua |  |  |  | 1 |  | 1 |  |  |
| Dasypodidae |  |  |  |  |  |  |  |  |  |
| *Priodontes maximus* | giant armadillo | 4 | 3 | 8 | 4 | 3 | 3 | 5 | 3 |
| *Dasypus novemcinctus* | nine-banded armadillo | 17 | 25 | 37 | 29 | 60 | 12 | 31 | 39 |
| Callitrichidae |  |  |  |  |  |  |  |  |  |
| *Saguinus tripartitus* | golden-mantle tamarin |  | 1 |  |  |  |  |  |  |
| Cebidae |  |  |  |  |  |  |  |  |  |
| *Ateles belzebuth* | white-bellied spider  monkey |  |  |  |  |  | 1 |  |  |
| *Cebus albifrons* | white-fronted  capuchin |  |  |  | 3 |  |  |  |  |
| Canidae |  |  |  |  |  |  |  |  |  |
| *Atelocynus microtis* | short-eared dog | 5 | 13 | 1 | 2 |  |  | 2 |  |
| Procyonidae |  |  |  |  |  |  |  |  |  |
| *Procyon cancrivorus* | crab-eating raccoon | 1 |  |  |  |  | 1 | 1 | 2 |
| *Nasua nasua* | South American coati | 1 | 1 | 2 | 2 | 6 | 1 | 2 | 2 |
| Mustelidae |  |  |  |  |  |  |  |  |  |
| *Eira barbara* | tayra |  |  | 1 | 1 | 3 |  |  | 1 |
| Felidae |  |  |  |  |  |  |  |  |  |
| *Leopardus pardalis* | ocelot | 4 | 8 | 3 | 9 | 22 | 8 | 5 | 19 |
| *Leopardus wiedii* | margay |  | 4 |  |  |  | 1 |  |  |
| *Puma concolor* | puma | 7 | 1 | 45 | 12 | 9 | 1 | 25 | 9 |
| *Puma yagouaroundi* | jaguarundi | 1 | 1 | 1 |  |  |  |  |  |
| *Panthera onca* | jaguar | 3 | 6 | 6 | 4 | 2 | 6 | 12 | 2 |
| Tapiridae |  |  |  |  |  |  |  |  |  |
| *Tapirus terrestris* | South American tapir | 11 | 8 | 12 | 12 | 49 | 71 | 34 | 53 |
| Tayassuidae |  |  |  |  |  |  |  |  |  |
| *Pecari tajacu* | collared peccary | 125 | 132 | 94 | 139 | 84 | 78 | 44 | 53 |
| *Tayassu pecari* | white-lipped peccary | 16 | 6 | 13 | 27 | 61 | 70 | 58 | 79 |
| Cervidae |  |  |  |  |  |  |  |  |  |
| *Mazama americana* | red brocket deer | 23 | 55 | 77 | 67 | 58 | 118 | 62 | 92 |
| *Mazama nemorivaga** | gray brocket deer | 7 | 2 | 9 | 1 |  |  | 3 | 1 |
| Erithozontidae |  |  |  |  |  |  |  |  |  |
| *Coenodou prehensilis* | prehensile-tailed  porcupine |  |  |  |  |  |  |  | 1 |
| Sciuridae |  |  |  |  |  |  |  |  |  |
| *Sciurus igniventris* | northern Amazon red  squirrel | 8 | 2 | 1 | 1 |  |  |  | 4 |
| Cuniculidae |  |  |  |  |  |  |  |  |  |
| *Cuniculus paca* | Paca | 51 | 30 | 28 | 31 | 15 | 27 | 26 | 37 |
| Dasyproctidae |  |  |  |  |  |  |  |  |  |
| *Dasyprocta fuliginosa* | black agouti | 38 | 39 | 37 | 26 | 63 | 53 | 14 | 67 |
| *Myoprocta pratti* | green acouchy | 13 | 21 | 23 | 9 | 102 | 122 | 74 | 62 |
| Echimyidae |  |  |  |  |  |  |  |  |  |
| *Proechimys* sp. | spiny rat |  | 2 |  | 1 | 1 |  |  | 3 |
|  |  |  |  |  |  |  |  |  |  |
