# Supplementary material for: Annual and spatial variation in composition and activity of terrestrial mammals on two replicate plots in lowland forest of eastern Ecuador
Source: PeerJ. 2018 Jan 9;6:e4241. doi: 10.7717/peerj.4241 (PMC5765811; doi:10.7717/peerj.4241)
Supplement: Table S2 — Values (mean, SE) of variables used to characterize environmental conditions within either 50 m or 100 m radius of camera locations on two plots in lowland forest at Tiputini Biodiversity Station, Ecuador. Values are given for both plots combined and for each plot separately. Comparisons between plots were based on two-sample t-tests. [file peerj-06-4241-s004.docx]

| Variable | Combined plots | | Harpia plot | | Puma plot | |  |  |
| --- | --- | --- | --- | --- | --- | --- | --- | --- |
|  | Mean | SE | Mean | SE | Mean | SE | test stat | *P* = |
| **50 m buffer** |  |  |  |  |  |  |  |  |
| Elevation | 217 | 0.79 | 217 | 1.33 | 216 | 0.88 |  |  |
| Elevation SD | 2.40 | 0.19 | 2.86 | 0.26 | 1.94 | 0.21 | *t* = 2.74 | 0.01 |
| Slope | 8.43 | 0.47 | 9.10 | 0.63 | 7.77 | 0.68 | *t* = 1.43 | 0.16 |
| Slope SD | 5.38 | 0.39 | 5.16 | 0.46 | 5.61 | 0.64 | *t* = 0.57 | 0.57 |
| Distance to stream | 22.1 | 1.89 | 25.2 | 2.61 | 19.1 | 2.58 | *t* = 1.67 | 0.10 |
| Distance to stream SD | 12.6 | 0.66 | 13.9 | 0.91 | 11.2 | 0.86 | *t* = 2.14 | 0.04 |
| **100 m buffer** |  |  |  |  |  |  |  |  |
| Elevation | 216 | 0.64 | 215 | 1.11 | 216 | 0.70 |  |  |
| Elevation SD | 3.53 | 0.20 | 4.25 | 0.22 | 2.81 | 0.22 | *t* = 4.63 | 0.001 |
| Slope | 8.12 | 0.34 | 9.04 | 0.36 | 7.19 | 0.47 | *t* = 3.10 | 0.004 |
| Slope SD | 5.75 | 0.28 | 5.66 | 0.32 | 5.82 | 0.47 |  |  |
| Distance to stream* | 23.0 | 1.15 | 25.2 | 1.53 | 20.8 | 1.59 | *t* = 2.19 | 0.04 |
| Distance to stream SD* | 16.4 | 0.70 | 16.9 | 0.86 | 15.9 | 1.12 | *t* = 0.90 | 0.38 |
